# Conservative treatment of acute limb ischemia in a child - case report

**DOI:** 10.1590/1677-5449.202200101

**Published:** 2022-11-14

**Authors:** Suzanna Maria Viana Sanches, Maurício de Amorim Aquino, Brenda de Lima Leite, Monique Magnavita Borba da Fonseca Cerqueira

**Affiliations:** 1 Santa Casa da Bahia, Salvador, BA, Brasil.; 2 Rede D’Or, Centro de Hematologia e Oncologia da Bahia, Salvador, BA, Brasil.; 3 Universidade Federal da Bahia - UFBA, Hospital Universitário Professor Edgard Santos, Salvador, BA, Brasil.; 4 Hospital Ana Nery - HAN, Salvador, BA, Brasil.; 5 Universidade do Estado da Bahia - UNEB, Salvador, BA, Brasil.

**Keywords:** ischemia, children, anticoagulants, case report

## Abstract

Acute limb ischemia is a rare event in the pediatric population, with an estimated incidence of 26 per 100,000 admissions, the majority of which are associated with trauma or iatrogeny. The ideal treatment for these cases is not well-established in the literature, although there is a tendency to choose noninvasive treatment. We report the case of an infant who suffered ischemia to all four limbs secondary to hemodynamic complications after complex heart surgery and in whom significant tissue preservation was achieved with conservative treatment.

## INTRODUCTION

Acute limb ischemia is sudden interruption of blood flow to an extremity, resulting in insufficient supply of oxygen to tissues. If appropriate revascularization is not performed, the condition can progress to irreversible damage to structures after 4 to 6 hours.^1^ It is a rare, but devastating complication in the pediatric population, with a rate of occurrence of 26 cases per 100,000 admissions.^2^

Within this age group, acute ischemia is most often caused by trauma or iatrogeny, although it can occasionally be the consequence of thromboembolic events, as occurs in adults.^3^ The rarity of these events in the infant population means that there are few data on the ideal treatment to achieve limb salvage and prevent morbidity.

The objective of this report is to document the satisfactory outcome of a case of acute limb ischemia in an infant that was caused by hypoperfusion secondary to hemodynamic instability during the perioperative period of complex heart surgery and was managed with conservative treatment. The study was approved in advance by the Ethics Committee (Ethics Review Submission Certificate [CAAE]: 54149121.7.0000.5520; decision number: 5.194.867).

## CASE REPORT

The case involved a female infant, diagnosed at birth with congenital heart disease consisting of interatrial and interventricular communication and persistent ductus arteriosus. At 3 months of age, she underwent a surgical procedure to treat her condition, with pulmonary artery banding and clipping of the ductus arteriosus. At 23 months of age, she underwent another procedure, to perform atrial septoplasty, ventricular septoplasty, and removal of the pulmonary artery banding.

Intraoperatively, during the second intervention, the patient suffered cardiorespiratory arrest lasting 4 minutes, before extracorporeal circulation (ECC) was initiated. This complication was caused by hemorrhagic shock secondary to laceration of the right atrium wall, which in turn was a complication of lysing adherences during surgical dissection. Multiple transfusions and administration of vasoactive amines in moderate doses were needed to control shock. The total duration of ECC recorded was 3 hours and 45 minutes, with anoxia lasting 2 hours.

After the procedure, the infant was admitted to the pediatric intensive care unit (ICU) in a hemodynamically instable state. At this point she was hypotense, with weak pulses, cold extremities, capillary refill time (CRT) of 4 seconds, and hyperlactatemia. She was monitored with invasive arterial blood pressure monitoring via the right radial artery.

She exhibited worsening of cardiogenic shock, with signs of low output and deterioration of perfusion, her CRT increased to 8 seconds, and serum lactate reached 6.0 mmol/L. Physical examination found her extremities cold, from the forearm and leg. At this point, she was on adrenaline (2 mcg/kg/min), noradrenaline (2.0 mcg/kg/min), milrinone (0.7 mcg/kg/min), dobutamine (10 mcg/kg/min), and vasopressin (0.4 UI/kg/h).

Low blood flow rate, peripheral vasoconstriction, cardiogenic shock, and the consequent need for vasoactive drugs to treat hemodynamic instability worsened the perfusion to the infant’s extremities over the following days, resulting in cyanosis of the fingers of both hands, worse on the left, and the toes of both feet, more intensely on the right. At this point she was sedated, intubated, and on mechanical ventilation, and no signs of pain were recorded.

**Figure 1:**
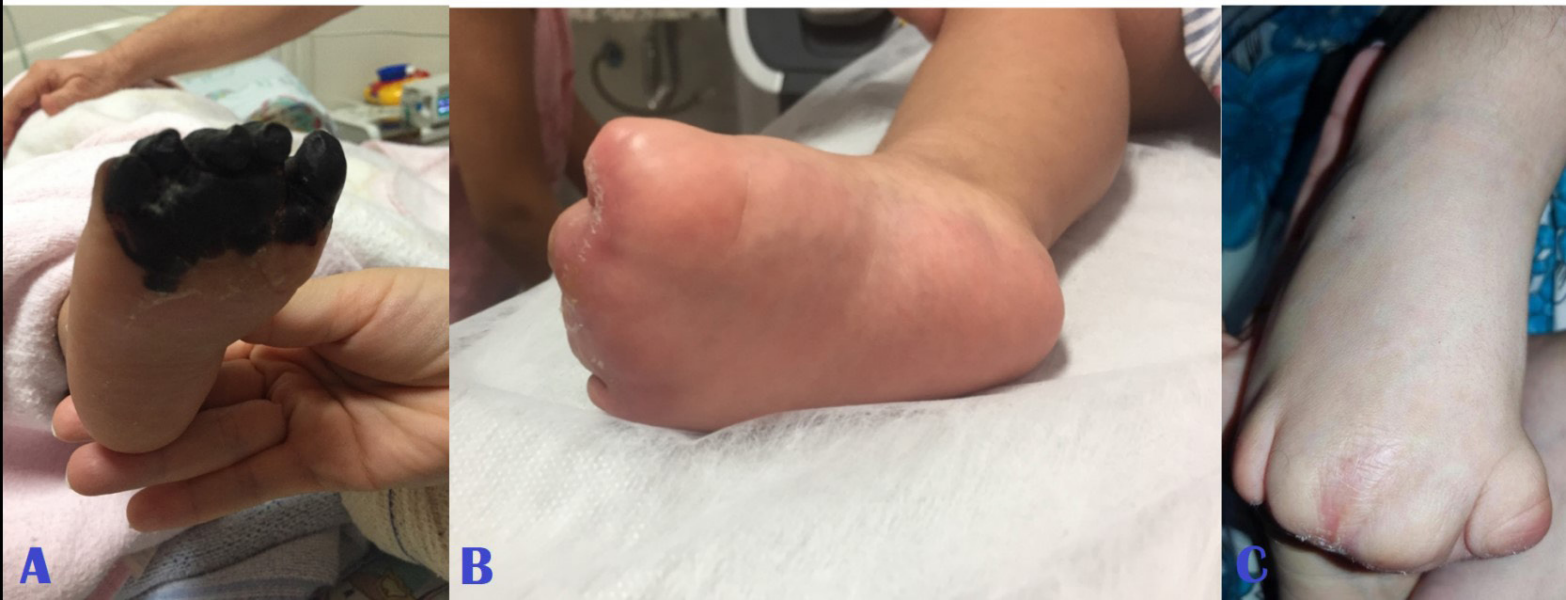
**(A)** Right foot (appearance in July/2018); (**B)** right foot (appearance in December/2018) with no surgical intervention; (**C)** right foot (current appearance, 4 years after the initial examination).

The patient was seen by the vascular surgery team, by which time she was breathing spontaneously again and on systemic analgesia. They found palpable distal pulses in the upper and lower limbs and observed that motricity of the extremities was maintained. They prescribed mechanical protection of the limbs against heat loss and full anticoagulation with enoxaparin at a dosage of 1 mg/kg every 12 hours until the areas of irreversible ischemia were delimited. Over the days that followed, she developed areas of dry necrosis involving all four extremities (Figures 1 [Fig gf0200] [Fig gf0300]-4, item A).

**Figure 2 gf0200:**
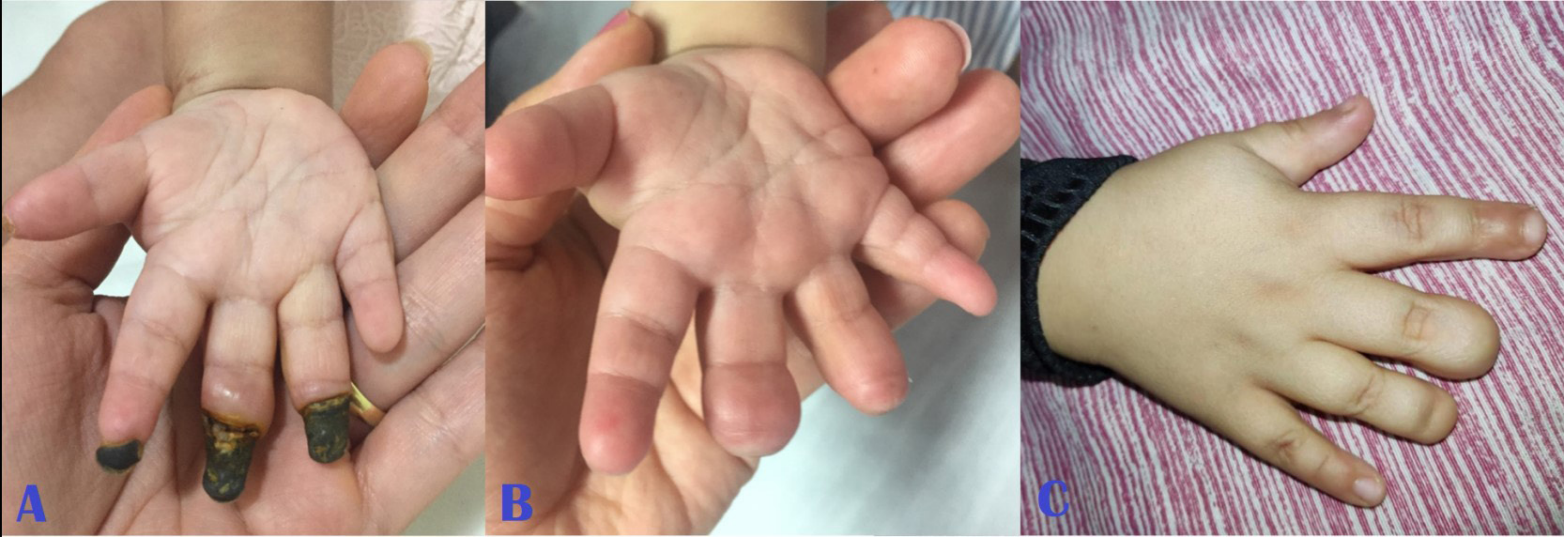
**(A)** Right hand (appearance in August/2018); (**B)** right hand (appearance in December/2018) with no surgical intervention; (**C)** right hand (current appearance, 4 years after the initial examination).

**Figure 3 gf0300:**
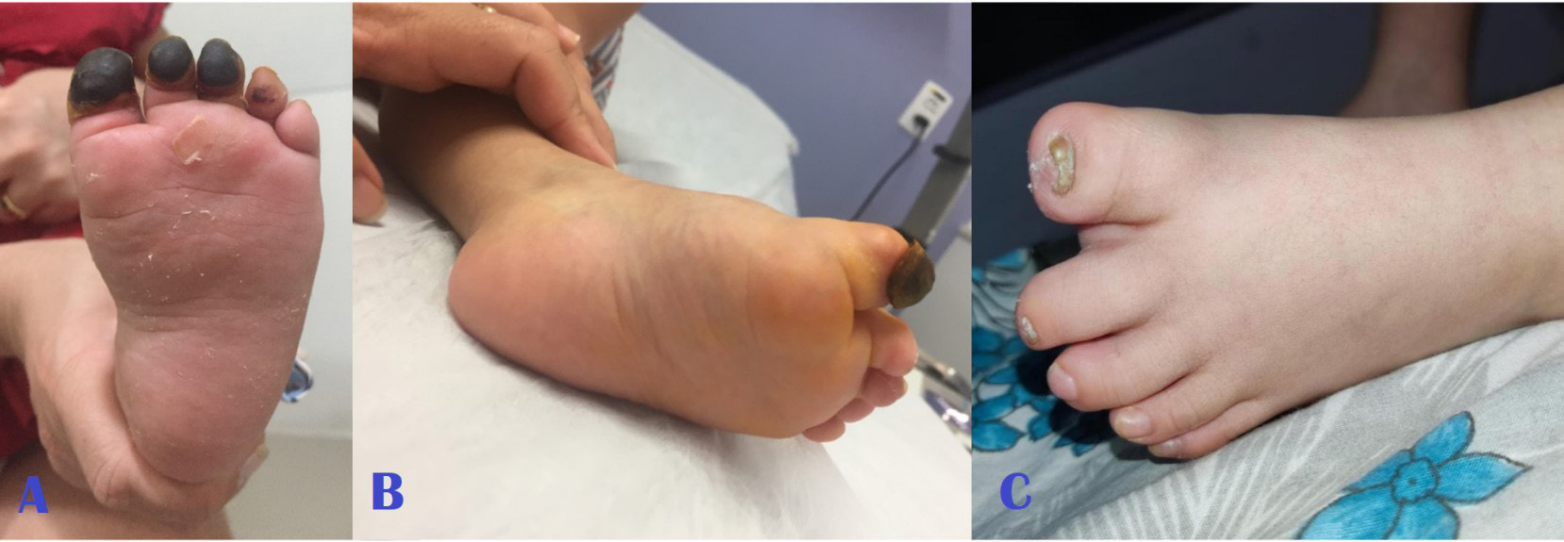
**(A)** Left foot (appearance in July/2018); (**B)** left foot (appearance in December/2018) with no surgical intervention; (**C)** left foot (current appearance, 4 years after the initial examination).

**Figure 4:**
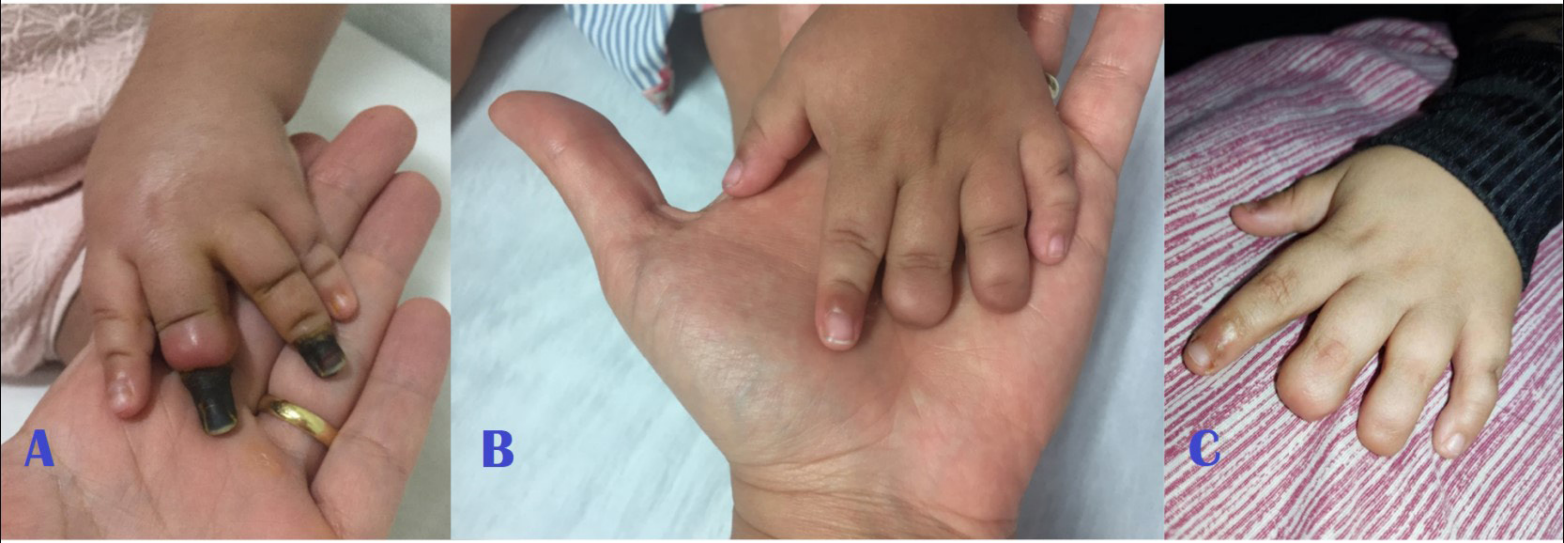
**(A)** Left hand (appearance in August/2018); (**B)** left hand (appearance in December/2018) with no surgical intervention; (**C)** left hand (current appearance, 4 years after the initial examination).

In view of the extent of the injuries and the risk of infection of nonviable tissues, amputation of the areas of delimited necrosis was indicated. However, the child’s family did not give consent for extensive mutilatory surgery and in conjunction with the treating team the decision was taken to maintain conservative treatment with regular outpatients follow-up after discharge from hospital.

During follow-up, there was no need to administer analgesics, because the child did not complain of pain. The full anticoagulation initiated in hospital was continued for 3 months. Over this period, ischemia improved, with significant regression of areas previously considered to have suffered irreversible tissue damage, with necrosis restricted to the distal portions of fingers and toes (Figures 1-4, item B).

During the second month of follow-up, the child exhibited inflammatory signs compatible with infections of the third finger of both hands. She was treated with oral antibiotics, which were combined with hyperbaric oxygen therapy sessions - which had been recommended by the pediatrics team at a different center. The current appearance of her extremities is shown in Figures 1-4, item C.

## DISCUSSION

Limb ischemia in children should be interpreted as a distinct nosological entity from atherosclerotic or thrombotic arterial events seen in adults, because of the differences in etiology, physiology, and prognosis. These conditions tend to be caused by congenital, iatrogenic, traumatic, or infectious factors. There are also reports of associations with Kawasaki disease.^4^^,^^5^ In babies, iatrogenic cases are more common, while trauma becomes the predominant cause in children and adolescents.^3^

Direct or inadvertent cannulation of arteries during diagnostic and therapeutic interventions is the number one iatrogenic cause of ischemia in children, and only a small proportion of clinical presentations of this type of complication involve complete interruption of blood flow. In these cases, the limb may be sustained by small preexisting trunk vessels that remain healthy or by early collateralization, without requiring surgical intervention to restore flow.^6^

It is known that children’s arteries have thin walls and a tendency to intense vasospasm after manipulation, which makes vascular instrumentation challenging. The results of revascularization are poor in pediatric patients, with mortality estimated at 14%.^7^ The physiological response to ischemia also tends to be different, because there is no atherosclerotic involvement, compensation by ischemia-induced vasodilation tends to be less significant, and there is a smaller quantity of muscle mass, which appears to contribute to greater tolerance of ischemia.^2^

Since these events are rare in the pediatric population, there are few published data on the ideal management to achieve limb salvage and prevent morbidity.^2^^,^^8^ Treatment strategies have been chosen on the basis of extrapolation of data from treatment of adult patients, because no prospective studies with appropriate designs have been conducted with pediatric populations.^1^

Previously published studies suggest that anticoagulation is beneficial in ischemic thrombotic conditions; but because of the lack of data, pediatricians and surgeons do not have guidelines that define the ideal management for limb salvage in children.^1^ For example, type of anticoagulant, dosage, and even duration of anticoagulation are not well-documented. The majority of events reported were treated with unfractionated heparin or low molecular weight heparin, for periods ranging from 1 day to 3.9 months.^1^^,^^3^^,^^6^ The risk of secondary infection is described as high^9^ and some reports also mention antiplatelet aggregation as an adjuvant strategy.^10^

In cases of peripheral ischemia secondary to vasculitis, such as in Kawasaki disease, in addition to antiplatelet aggregation with aspirin, some studies also describe administration of intravenous immunoglobulin and prostaglandin E1.^4^^,^^5^ However, to date, there is no robust, quality scientific evidence to support these practices, so more studies are needed to evaluate the risks and benefits.

## CONCLUSIONS

The case described illustrates the benefits of conservative management in an infant with ischemia of the limbs after shock. Considering the characteristics of the pediatric population, when faced with ischemia in this population, it is known that any premature procedures could provoke complications, with a risk of limb loss, so if there are no contraindications, conservative treatment may be the best option.^10^

In view of the tendency to severity in cases of children with arterial ischemia and the frequent doubt with regard to the ideal treatment, it is necessary to conduct studies with appropriate designs to evaluate the possible strategies for management of these situations.
