# Supplementary material for: Conservative treatment of acute limb ischemia in a child - case report
Source: J Vasc Bras. 2022 Nov 14;21:e20220010. doi: 10.1590/1677-5449.202200101 (PMC9675055; doi:10.1590/1677-5449.202200101)
Supplement: Translation

# Translation: Tratamento conservador de isquemia aguda de extremidades em lactente - relato de caso

**DOI:** 10.1590/1677-5449.202200102

**Authors:** Suzanna Maria Viana Sanches, Maurício de Amorim Aquino, Brenda de Lima Leite, Monique Magnavita Borba da Fonseca Cerqueira

**Affiliations:** 1 Santa Casa da Bahia, Salvador, BA, Brasil.; 2 Rede D’Or, Centro de Hematologia e Oncologia da Bahia, Salvador, BA, Brasil.; 3 Universidade Federal da Bahia - UFBA, Hospital Universitário Professor Edgard Santos, Salvador, BA, Brasil.; 4 Hospital Ana Nery - HAN, Salvador, BA, Brasil.; 5 Universidade do Estado da Bahia - UNEB, Salvador, BA, Brasil.

**Keywords:** isquemia, crianças, anticoagulantes, relato de caso

## Abstract

A isquemia aguda de extremidades é um evento raro na população pediátrica, com incidência estimada em 26 a cada 100.000 internações, sendo a maioria associada a trauma ou iatrogenia. O tratamento ideal para esses quadros não está bem estabelecido pela literatura, havendo a tendência ao tratamento não invasivo. Relatamos o caso de uma lactente que apresentou isquemia dos quatro membros secundária a complicações hemodinâmicas após realização de cirurgia cardíaca complexa, apresentando preservação tecidual expressiva com o tratamento conservador.

## INTRODUÇÃO

A isquemia aguda de extremidades consiste na interrupção repentina do fluxo sanguíneo, levando ao suprimento inadequado de oxigênio para os tecidos. Essa condição pode evoluir para lesão irreversível de estruturas após 4 a 6 horas, caso não seja realizada revascularização apropriada^1^. É uma complicação rara, porém devastadora na população pediátrica, com taxa de ocorrência de 26 casos para cada 100.000 internações^2^.

Nesse segmento etário, a isquemia aguda é mais comumente causada por trauma ou iatrogenia, sendo esporadicamente consequência de eventos tromboembólicos, como ocorre em adultos^3^. Em virtude da raridade dessas ocorrências na população infantil, há poucos dados sobre o tratamento ideal para salvamento de membros e prevenção de morbidade.

O objetivo deste relato foi documentar a evolução satisfatória de um caso de isquemia aguda das extremidades de uma lactente, ocorrida em função de hipoperfusão secundária à instabilidade hemodinâmica, no perioperatório de cirurgia cardíaca complexa, mediante tratamento conservador. O estudo foi realizado após aprovação pelo Comitê de Ética (Certificado de Apresentação de Apreciação Ética [CAAE]: 54149121.7.0000.5520, número do parecer: 5.194.867).

## RELATO DE CASO

O caso tratava-se de uma lactente do sexo feminino, diagnosticada na ocasião do seu nascimento com cardiopatia congênita, apresentando quadro de comunicação interatrial e interventricular, além de persistência do canal arterial. Para o tratamento da sua condição, aos 3 meses de idade, foi submetida a procedimento cirúrgico com realização de cerclagem da artéria pulmonar e clipagem de canal arterial. Aos 23 meses de idade, foi submetida a novo procedimento para atriosseptoplastia, ventriculosseptoplastia e retirada de cerclagem da artéria pulmonar.

No intraoperatório da segunda abordagem cirúrgica, apresentou parada cardiorrespiratória com duração de 4 minutos, antes do início da circulação extracorpórea (CEC). A complicação aconteceu por quadro de choque hemorrágico após laceração da parede do átrio direito, como complicação da lise de aderências durante a dissecção cirúrgica. Para controle do choque, foi necessária a realização de politransfusão e administração de aminas vasoativas em doses moderadas. O tempo total relatado de CEC foi de 3 horas e 45 minutos, com anoxia durante 2 horas.

Após o procedimento, a lactente foi admitida na unidade de terapia intensiva (UTI) pediátrica, apresentando instabilidade hemodinâmica. Na ocasião, encontrava-se hipotensa, com pulsos finos, extremidades frias, tempo de enchimento capilar (TEC) de 4 segundos e hiperlactatemia. Para monitorização, foi utilizada medida de pressão arterial invasiva na artéria radial direita.

Na evolução, cursou com piora do quadro de choque cardiogênico, mantendo sinais de baixo débito e piora da perfusão, com aumento do TEC para 8 segundos e com valor de lactato sérico de 6,0 mmol/L. Ao exame físico, apresentava frialdade nas extremidades, desde antebraço e perna. Neste momento, estava em uso de adrenalina (2 mcg/kg/min), noradrenalina (2,0 mcg/kg/min), milrinona (0,7 mcg/kg/min), dobutamina (10 mcg/kg/min) e vasopressina (0,4 UI/kg/h).

Devido ao quadro de hipofluxo, com vasoconstrição periférica pelo choque cardiogênico e com consequente necessidade do uso de drogas vasoativas para tratar a instabilidade hemodinâmica, a lactente evoluiu nos dias subsequentes com piora da perfusão das extremidades, apresentando cianose de quirodáctilos de ambas as mãos, mais acentuada à esquerda, e de pododáctilos bilateralmente, mais intensa à direita. Nesse momento, encontrava-se sedada e intubada, sob ventilação mecânica, não tendo sido reportada queixa de dor.

A paciente foi avaliada pela equipe da cirurgia vascular, já em ventilação espontânea sob analgesia sistemática, que identificou a presença de pulsos palpáveis distalmente em membros superiores e inferiores, além da preservação da motricidade das extremidades. Neste momento, o grupo orientou a proteção mecânica dos membros contra a perda de calor, associada à anticoagulação plena com enoxaparina na dose de 1 mg/kg a cada 12 horas até que houvesse a delimitação das áreas de isquemia irreversível. Evoluiu nos dias subsequentes com áreas de necrose seca nas quatro extremidades (Figuras 1 [Fig gf02] [Fig gf03]-4, item A).

**Figura 1:** **(A)** Pé direito (aspecto em julho/2018); (**B)** pé direito (aspecto em dezembro/2018) sem intervenção cirúrgica; (**C)** pé direito (aspecto atual, 4 anos após a avaliação inicial).

**Figura 2 gf02:** **(A)** Mão direita (aspecto em agosto/2018); (**B)** mão direita (aspecto em dezembro/2018) sem intervenção cirúrgica; (**C)** mão direita (aspecto atual, 4 anos após a avaliação inicial).

**Figura 3 gf03:** **(A)** Pé esquerdo (aspecto em julho/2018); (**B)** pé esquerdo (aspecto em dezembro/2018) sem intervenção cirúrgica; (**C)** pé esquerdo (aspecto atual, 4 anos após a avaliação inicial).

**Figura 4:** **(A)** Mão esquerda (aspecto em agosto/2018); (**B)** Mão esquerda (aspecto em dezembro/2018) sem intervenção cirúrgica; (**C)** Mão esquerda (aspecto atual, 4 anos após a avaliação inicial).

Em virtude da extensão das lesões e do risco de infecção dos tecidos inviáveis, foi indicada amputação das áreas de necrose já delimitadas. No entanto, a família não consentiu com a realização de uma cirurgia mutiladora extensa, tendo optado em conjunto com a equipe pela manutenção do tratamento conservador, com seguimento ambulatorial regular após a alta hospitalar.

Durante o acompanhamento, não houve a necessidade de uso de analgésicos, pois a criança não manifestou queixas de dor. A anticoagulação plena estabelecida durante o internamento foi mantida por 3 meses. Na evolução, observou-se a melhora do quadro de isquemia, com regressão importante das áreas previamente consideradas com dano tecidual irreversível, tendo a necrose ficado restrita às porções distais dos dedos (Figuras 1-4, item B).

Por volta do segundo mês de acompanhamento, a criança apresentou alterações flogísticas compatíveis com quadro infeccioso no terceiro quirodáctilo de ambas as mãos. Foi tratada com antibióticos via oral, tendo sido o tratamento associado a sessões de oxigenoterapia hiperbárica - uma orientação da equipe de pediatria de outro serviço. O aspecto atual das extremidades está evidenciado nas Figuras 1-4, item C.

## DISCUSSÃO

A isquemia de membro em crianças deve ser interpretada como entidade nosológica distinta dos eventos arteriais ateroscleróticos ou trombóticos vistos em adultos, em virtude das diferenças etiológicas, fisiológicas e relativas ao prognóstico. Essas condições costumam ser causadas por fatores congênitos, iatrogênicos, traumáticos ou infecciosos. Há ainda relatos associados à doença de Kawasaki^4^^,^^5^. Em bebês, as iatrogenias são mais comuns, enquanto o trauma se insinua como causa predominante em crianças e adolescentes^3^.

A canulação direta ou inadvertida de artérias durante intervenções diagnósticas e terapêuticas é a principal causa iatrogênica de isquemia em crianças, sendo que a completa interrupção do fluxo sanguíneo representa uma pequena parcela das apresentações clínicas desse tipo de complicação. Nesses casos, a manutenção do membro pode ser viabilizada por pequenos troncos saudáveis preexistentes ou por colateralização precoce, sem necessidade de uma intervenção cirúrgica restauradora do fluxo^6^.

Sabe-se que as artérias de crianças são caracterizadas por parede delgada e tendência a vasoespasmo intenso após manipulação, o que torna a instrumentalização vascular desafiadora. Os resultados associados a revascularização em pacientes pediátricos são ruins, com mortalidade estimada em 14%^7^. A resposta fisiológica à isquemia também costuma ser diferente, pela ausência de acometimento aterosclerótico e melhor compensação pela vasodilatação induzida por isquemia, além da menor quantidade de massa muscular, o que parece contribuir para maior tolerância à isquemia^2^.

Em virtude da raridade desses eventos na população infantil, há poucos dados sobre o manejo ideal para salvamento de membros e prevenção de morbidade^2^^,^^8^. As estratégias de tratamento têm sido baseadas em extrapolações de dados do tratamento do paciente adulto, em função da ausência de trabalhos prospectivos, com desenho adequado, na população pediátrica^1^.

Estudos previamente publicados sugeriram benefício da anticoagulação em quadros isquêmicos trombóticos; contudo, em virtude da escassez de dados, pediatras e cirurgiões não dispõem de diretrizes que definam o manejo ideal para salvamento do membro em crianças^1^. Não está bem documentada, por exemplo, a conduta referente ao tipo de anticoagulante, dose ou mesmo tempo de anticoagulação. A maioria dos eventos relatados foi tratada com heparina não fracionada ou de baixo peso molecular por períodos variados entre 1 dia e 3,9 meses^1^^,^^3^^,^^6^. O risco de infecção secundária é descrito como alto9, e alguns relatos citam, ainda, o uso de antiagregação plaquetária como estratégia adjuvante^10^.

Em casos de isquemia periférica secundária a vasculite, como na doença de Kawasaki, estudos relatam, além da antiagregação plaquetária com aspirina, a administração de imunoglobulina intravenosa e prostaglandina E1^4^^,^^5^. No entanto, não há, até o presente momento, evidências científicas robustas e de qualidade que possam nortear essas práticas, sendo necessários mais estudos para avaliar riscos e benefícios.

## CONCLUSÕES

O caso relatado ilustra o benefício do manejo conservador em uma lactente com isquemia de extremidades após quadro de choque. Considerando as características da população pediátrica, diante de quadros isquêmicos nessa população, sabe-se que qualquer procedimento intempestivo pode levar a complicações, com risco de perda de extremidade; desse modo, não havendo contraindicações, o tratamento conservador pode ser a melhor conduta a ser adotada^10^.

Diante da gravidade das crianças usualmente acometidas por isquemia arterial e das frequentes dúvidas com relação ao tratamento ideal, torna-se necessária a realização de estudos com desenho adequado para avaliar as possíveis estratégias de abordagem nessas situações.
